# Supplementary material for: B cell heterogeneity in human tuberculosis highlights compartment-specific phenotype and functional roles
Source: Commun Biol. 2024 May 16;7:584. doi: 10.1038/s42003-024-06282-7 (PMC11099031; doi:10.1038/s42003-024-06282-7)
Supplement: Supplementary file 2 — Supplementary information [file 42003_2024_6282_MOESM2_ESM.pdf]

Supplementary information

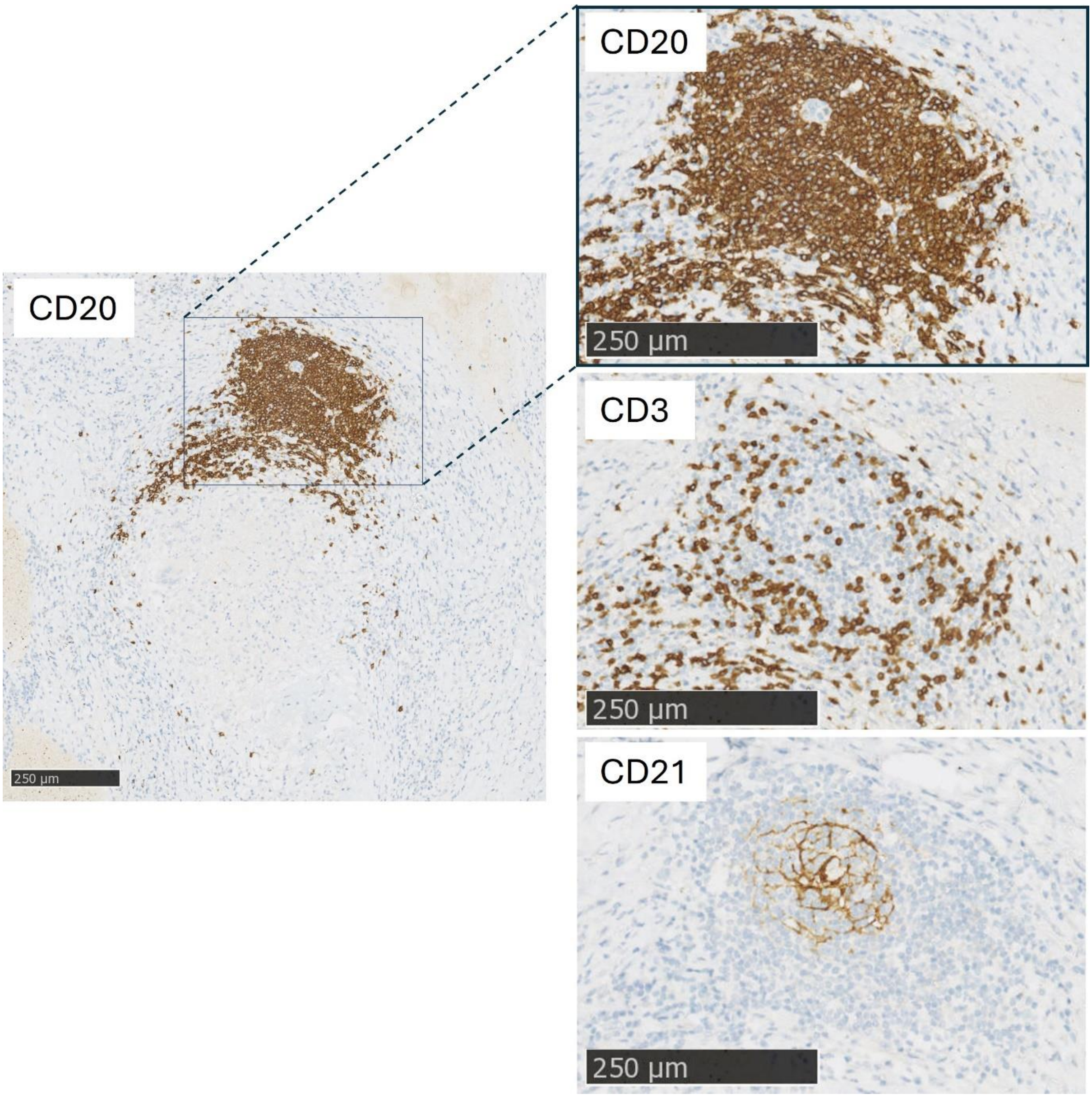

Supplementary Figure 1: Immunohistochemistry of human lung tissue resection from a TB patient demonstrates cellular organisation within aggregates associated with the granuloma. Serial sections of TB patient lung tissue were stained for B cells (anti-CD20), T cells (anti-CD3) and dendritic cells/mature B cells (anti-CD21). The boxed region is enlarged to illustrate the distinct staining patterns of the different markers within the granuloma associated aggregates.

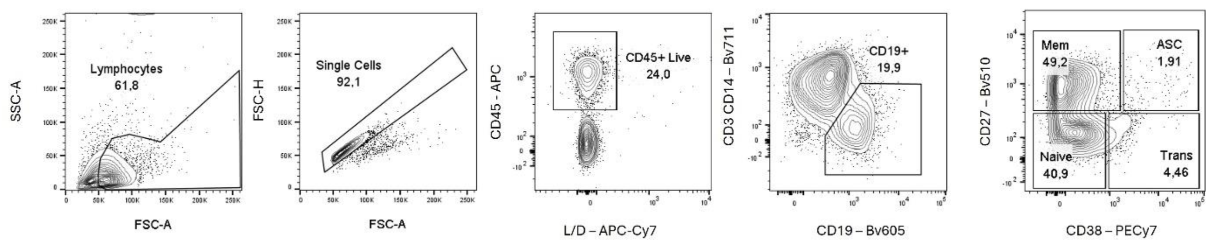

Supplementary Figure 2: Gating strategy to identify CD19^{+} B cells associated with the lung draining lymph node. Representative flow plots identifying CD19^{+} B cells derived from the lung draining lymph node of TB patient lungs. This was followed by gating the canonical transitional, naïve, memory and ASC phenotypes derived from staining the B cells with CD27 and CD38.

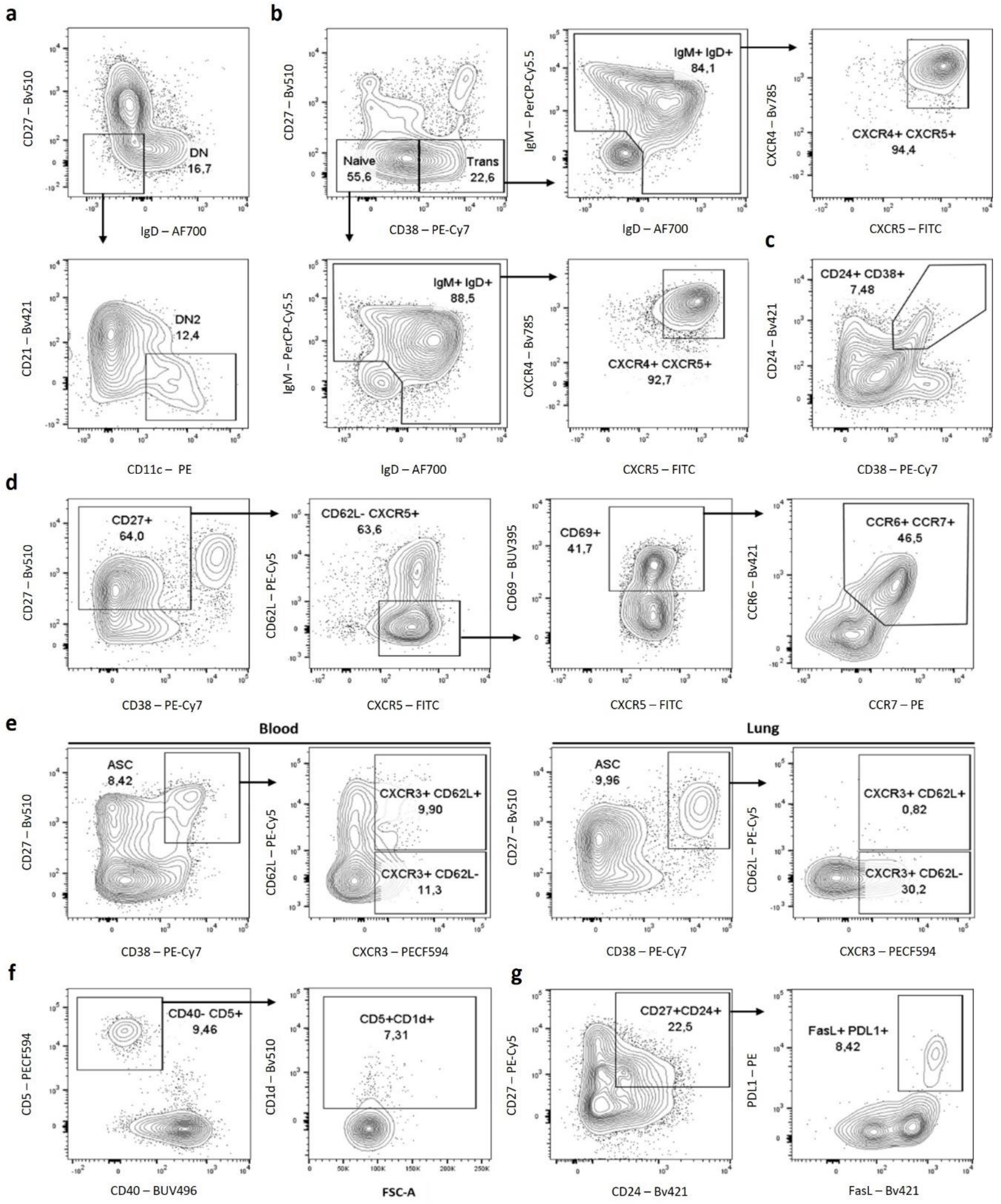

Supplementary Figure 3: Gating strategies for t-SNE derived B cell phenotypes associated with the blood or lung compartment. (**a**) Double negative (DN, CD27- IgD-) and activated DN (CD21- CD11c+) B cells. (**b**) Blood derived GC homing (CXCR4+ CXCR5+) transitional and naïve B cells. (**c**) Blood enriched tranistional regulatory (CD24+ CD38+) B cells. (**d**) The lung derived activated B cell memory (CD27+ CXCR5+ CD62L- CD69+ CCR6+ CCR7+) population. (**e**) Respective Blood and Lung derived ASC populations expressing a CXCR3 and/or CD62L. (**f**) A lung enriched CD5+ phenotype (CD5^{hi} CD40- CD1d+) and (**g**) a CD27+ regulatory population (CD27+ CD24+ FasL+ PDL1+).

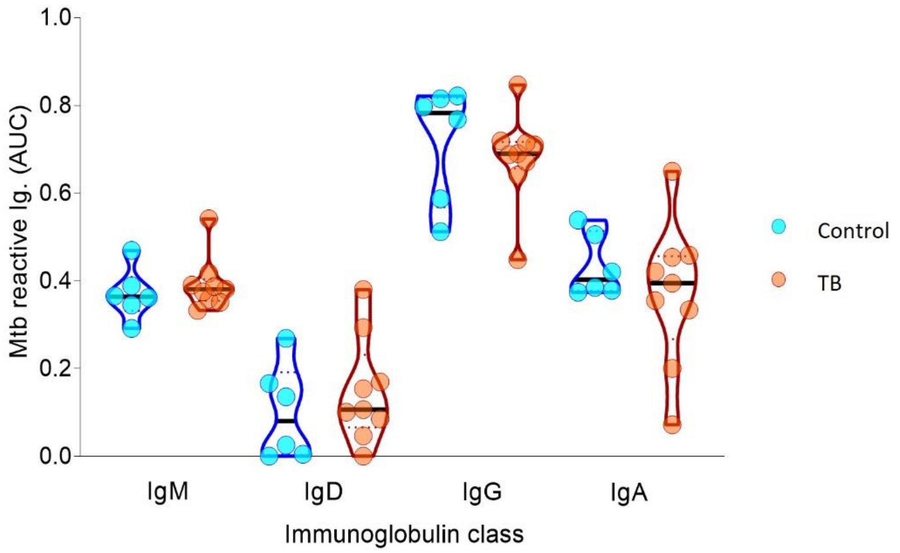

Supplementary Figure 4: *Mtb* specific response in patient plasma derived antibodies by ELISA. The *Mtb* specific antibody responses from cancer control (n=6) and TB patient (n=9) plasma, detecting class-specific responses. There were no significant differences as compared by Mann-Whitney test.

Supplementary Table 1: B cell maturation panel

Marker	Fluorochrome	Clone	Cat. no	Supplier	Dilution factor	Host
L/D	APC-Cy7		L10119	Invitrogen	200	
CD45	APC	HI30	304012	BioLegend	25	mouse
CD3	Bv711	OKT3	317328	BioLegend	50	mouse
CD14	Bv711	M5E2	301838	BioLegend	25	mouse
CD19	Bv605	HIB19	302244	BioLegend	50	mouse
CD27	Bv510	O323	302836	BioLegend	50	mouse
CD38	PECy7	HIT2	303516	BioLegend	50	mouse
IgM	PerCP/Cy5.5	MHM-88	314512	BioLegend	100	mouse
IgD	AF700	IA6-2	348230	BioLegend	25	mouse
CD138	Bv785™	MI15	356538	BioLegend	25	mouse
CXCR5	AF488	RF8B2	558112	BD Pharmingen	100	rat
CD11c	PE	S-HCL-3	371504	BioLegend	25	mouse
CD95 (Fas)	Bv650™	DX2	305642	BioLegend	50	mouse
CD20	PE/Dazzle™ 594	2H7	302348	BioLegend	50	mouse
CD69	BUV395	FN50	564364	BD Horizon	100	mouse
CD10	PE-Cy5	HI10a (RUO)	555376	BD Pharmingen	10	mouse
CD21	Bv421	B-ly4	562966	BD Horizon	100	mouse
CD40	BUV496	5C3	741159	BD OptiBuild	50	mouse

Supplementary Table 2: B cell homing panel

Marker	Fluorochrome	Clone	Cat. no	Supplier	Dilution factor	Host
L/D	APC-Cy7		L10119	Invitrogen	200	
CD45	APC	HI30	304012	BioLegend	25	mouse
CD3	Bv711	OKT3	317328	BioLegend	50	mouse
CD14	Bv711	M5E2	301838	BioLegend	25	mouse
CD19	Bv605	HIB19	302244	BioLegend	50	mouse
CD27	Bv510	O323	302836	BioLegend	50	mouse
CD38	PECy7	HIT2	303516	BioLegend	50	mouse
IgM	PerCP/Cy5.5	MHM-88	314512	BioLegend	100	mouse
IgD	AF700	IA6-2	348230	BioLegend	25	mouse
CCR6 (CD196)	Bv421	GO34E3	353439	BioLegend	25	mouse
CXCR5	AF488 (FITC)	RF8B2	558112	BD Pharmingen	100	rat
CXCR4 (CD184)	Bv785™	12G5	306530	BioLegend	50	mouse
CD62L	PE-Cy5	DREG-56	555545	BD Pharmingen	50	mouse
CXCR3 (CD183)	PE-CF594	IC6/CXCR3	562451	BD Horizon	25	mouse
CD69	BUV395	FN50	564364	BD Horizon	100	mouse
CCR7	PE	150503	FAB197P	R&D Biosystems	25	mouse

Supplementary Table 3: B cell regulatory panel

Marker	Fluorochrome	Clone	Cat. no	Supplier	Dilution factor	Host
L/D	APC-Cy7		L10119	Invitrogen	200	
CD45	APC	HI30	304012	BioLegend	25	mouse
CD3	Bv711	OKT3	317328	BioLegend	50	mouse
CD14	Bv711	M5E2	301838	BioLegend	25	mouse
CD19	Bv605	HIB19	302244	BioLegend	50	mouse
CD38	PECy7	HIT2	303516	BioLegend	50	mouse
IgM	PerCP/Cy5.5	MHM-88	314512	BioLegend	100	mouse
IgD	AF700	IA6-2	348230	BioLegend	25	mouse
CD27	PE-Cy5	1A4CD27	6607107	Beckman Coulter	25	mouse
CD40	BUV496	5C3	741159	BD OptiBuild	50	mouse
PD-L1 (CD274)	PE	29E.2A3	329706	BioLegend	50	mouse
CD24	FITC	ML5	311104	BioLegend	25	mouse
CD178 (Fas-L)	Bv421™	NOK-1	306412	BioLegend	10	mouse
CD1d	Bv510™	51.1	350314	BioLegend	10	mouse
CD5	PE/Dazzle™ 594	L17F12	364012	BioLegend	50	mouse
CD86	Bv650™	IT2.2	305428	BioLegend	50	mouse
