# Supplementary material for: B cell heterogeneity in human tuberculosis highlights compartment-specific phenotype and functional roles
Source: Commun Biol. 2024 May 16;7:584. doi: 10.1038/s42003-024-06282-7 (PMC11099031; doi:10.1038/s42003-024-06282-7)
Supplement: Supplementary file 5 — Reporting Summary [file 42003_2024_6282_MOESM5_ESM.pdf]

nature portfolio

Corresponding author(s): Robert Krause

Last updated by author(s): Mar 6, 2024

Reporting Summary

Nature Portfolio wishes to improve the reproducibility of the work that we publish. This form provides structure for consistency and transparency in reporting. For further information on Nature Portfolio policies, see our Editorial Policies and the Editorial Policy Checklist.

Statistics

For all statistical analyses, confirm that the following items are present in the figure legend, table legend, main text, or Methods section.

n/a	Confirmed	
☐	☒	The exact sample size (*n*) for each experimental group/condition, given as a discrete number and unit of measurement
☐	☒	A statement on whether measurements were taken from distinct samples or whether the same sample was measured repeatedly
☐	☒	The statistical test(s) used AND whether they are one- or two-sided<br>*Only common tests should be described solely by name; describe more complex techniques in the Methods section.*
☒	☐	A description of all covariates tested
☒	☐	A description of any assumptions or corrections, such as tests of normality and adjustment for multiple comparisons
☐	☒	A full description of the statistical parameters including central tendency (e.g. means) or other basic estimates (e.g. regression coefficient) AND variation (e.g. standard deviation) or associated estimates of uncertainty (e.g. confidence intervals)
☐	☒	For null hypothesis testing, the test statistic (e.g. *F*, *t*, *r*) with confidence intervals, effect sizes, degrees of freedom and *P* value noted<br>*Give P values as exact values whenever suitable.*
☒	☐	For Bayesian analysis, information on the choice of priors and Markov chain Monte Carlo settings
☒	☐	For hierarchical and complex designs, identification of the appropriate level for tests and full reporting of outcomes
☒	☐	Estimates of effect sizes (e.g. Cohen's *d*, Pearson's *r*), indicating how they were calculated

Our web collection on statistics for biologists contains articles on many of the points above.

Software and code

Policy information about availability of computer code

Data collection	No software was used.
Data analysis	Prism version 9; GraphPad Software Inc., San Diego, CA, USA<br>FlowJo version 9.9.6 (Tree Star)<br>Drop-Seq Computation Protocol v2.0 (https://github.com/broadinstitute/Drop-seq)<br>Seurat R package v3.1.0 (https://satijalab.org/seurat/)

For manuscripts utilizing custom algorithms or software that are central to the research but not yet described in published literature, software must be made available to editors and reviewers. We strongly encourage code deposition in a community repository (e.g. GitHub). See the Nature Portfolio guidelines for submitting code & software for further information.

Data

Policy information about availability of data

All manuscripts must include a data availability statement. This statement should provide the following information, where applicable:

- Accession codes, unique identifiers, or web links for publicly available datasets
- A description of any restrictions on data availability
- For clinical datasets or third party data, please ensure that the statement adheres to our policy

The original contributions presented in the study are included in the article/Supplementary Material. Further inquiries can be directed to the corresponding author.

Research involving human participants, their data, or biological material

Policy information about studies with [human participants or human data](). See also policy information about [sex, gender (identity/presentation), and sexual orientation]() and [race, ethnicity and racism]().

Reporting on sex and gender	Not applicable.
Reporting on race, ethnicity, or other socially relevant groupings	Not applicable.
Population characteristics	Human patients undergoing thoracotomy or lung resection surgery for TB or non-TB associated pathologies.
Recruitment	Human patients undergoing thoracotomy or lung resection surgery for TB or non-TB associated pathologies gave written informed consent for a blood draw and tissue collection. The study protocol, data collection tools and associated consent forms were approved by the University of KwaZulu-Natal Biomedical Research Ethics Committee (BE 019/13). The CUBS study protocol for blood collection from healthy donors and patients with active TB was also approved (BE 022/13). Healthy blood donor study ethical approval was provided by the National Research Ethics Service Committee South Central — Southampton A, ref 13/SC/0043, Southampton, United Kingdom.
Ethics oversight	University of KwaZulu-Natal Biomedical Research Ethics Committee<br>National Research Ethics Service Committee South Central — Southampton A

Note that full information on the approval of the study protocol must also be provided in the manuscript.

Field-specific reporting

Please select the one below that is the best fit for your research. If you are not sure, read the appropriate sections before making your selection.

☒ Life sciences ☐ Behavioural & social sciences ☐ Ecological, evolutionary & environmental sciences

For a reference copy of the document with all sections, see [nature.com/documents/nr-reporting-summary-flat.pdf]()

Life sciences study design

All studies must disclose on these points even when the disclosure is negative.

Sample size	No sample size calculations were performed as the samples form part of ongoing recruitment cohorts and in most cases the samples were processed as fresh samples without cryo-preservation in order to ensure highest quality data. In addition, the cancer control lung samples are very rare samples and usually yield small unaffected tissue sections yielding fewer cells than the TB patient samples. Therefore as many as possible were included in these analyses, but due to their scarcity they could not be included throughout all study comparisons.
Data exclusions	No data were excluded. Sample sizes do vary in part as the analyses were adapted to address/investigate B cells populations of interest.
Replication	In the case of the patient lung and blood samples, multiple biological replicates were included in order to verify the significance of the B cell phenotypes and populations identified. In most cases there is a direct comparison of the lung with its matching blood compartment or alternate tissue compartment. With regards to the biomimetic 3D culture model, multiple PBMC and/or immunoglobulin donors were used and the experiments themselves were replicated a minimum of 3 times to ensure reproducibility.
Randomization	There was no randomization. Patient groups were classified in accordance with their TB status. In addition, most samples were collected with accompanying/matching blood samples.
Blinding	Blinding was not applied, except in the case of the 3D biomimetic model as parts of these experiments were conducted at the collaborative laboratory of Prof Paul Elkington.

Reporting for specific materials, systems and methods

We require information from authors about some types of materials, experimental systems and methods used in many studies. Here, indicate whether each material, system or method listed is relevant to your study. If you are not sure if a list item applies to your research, read the appropriate section before selecting a response.

Materials & experimental systems

n/a	Involved in the study
☐	☒ Antibodies
☒	☐ Eukaryotic cell lines
☒	☐ Palaeontology and archaeology
☒	☐ Animals and other organisms
☒	☐ Clinical data
☒	☐ Dual use research of concern
☒	☐ Plants

Methods

n/a	Involved in the study
☒	☐ ChIP-seq
☐	☒ Flow cytometry
☒	☐ MRI-based neuroimaging

Antibodies

Antibodies used

Flow cytometry:
L/D APC-Cy7 cat# L10119 Invitrogen
CD45 APC clone HI30 cat# 304012 BioLegend
CD3 Bv711 clone OKT3 cat# 317328 BioLegend
CD14 Bv711 clone M5E2 cat# 301838 BioLegend
CD19 Bv605 clone HIB19 cat# 302244 BioLegend
CD27 Bv510 clone O323 3cat# 02836 BioLegend
CD38 PECy7 clone HIT2 cat# 303516 BioLegend
IgM PerCP/Cy5.5 clone MHM-88 cat# 314512 BioLegend
IgD AF700 clone IA6-2 cat# 348230 BioLegend
CD138 Bv785™ clone MI15 cat# 356538 BioLegend
CXCR5 AF488 clone RF8B2 cat# 558112 BD Pharmingen
CD11c PE clone S-HCL-3 cat# 371504 BioLegend
CD95 (Fas) Bv650™ clone DX2 cat# 305642 BioLegend
CD20 PE/Dazzle™ 594 clone 2H7 cat# 302348 BioLegend
CD69 BUV395 clone FN50 cat# 564364 BD Horizon
CD10 PE-Cy5 clone HI10a (RUO) cat# 555376 BD Pharmingen
CD21 Bv421 clone B-ly4 cat# 562966 BD Horizon
CD40 BUV496 clone 5C3 cat# 741159 BD OptiBuild
CCR6 (CD196) Bv421 clone GO34E3 cat# 353439 BioLegend
CXCR5 AF488 (FITC) clone RF8B2 cat# 558112 BD Pharmingen
CXCR4 (CD184) Bv785™ clone 12G5 cat# 306530 BioLegend
CD62L PE-Cy5 clone DREG-56 cat# 555545 BD Pharmingen
CXCR3 (CD183) PE-CF594 clone IC6/CXCR3 cat# 562451 BD Horizon
CD69 BUV395 clone FN50 cat# 564364 BD Horizon
CCR7 PE clone 150503 cat# FAB197P R&D Biosystems
CD27 PE-Cy5 clone 1A4CD27 cat# 6607107 Beckman Coulter
CD40 BUV496 clone 5C3 cat# 741159 BD OptiBuild
PD-L1 (CD274) PE clone 29E.2A3 cat# 329706 BioLegend
CD24 FITC clone ML5 cat# 311104 BioLegend
CD178 (Fas-L) Bv421™ clone NOK-1 cat# 306412 BioLegend
CD1d Bv510™ clone 51.1 cat# 350314 BioLegend
CD5 PE/Dazzle™ 594 clone L17F12 cat# 364012 BioLegend
CD86 Bv650™ clone IT2.2 cat# 305428 BioLegend
Histology: CD20 (M0755-CD20cy-L26, DAKO), CD68 (ab192847, Abcam), CD45 (M0701-2B11+PD7/26, DAKO), CD3 (ab16669,Abcam) and CD21 (M0784-1F8, Dako).
Western blotting and ELISA: Goat anti-human IgD-HRPO (cat. no 2030-05, Southern Biotech), Goat anti-human IgA-HRPO (cat. no 2050-05, Southern Biotech), Donkey anti-human IgG-HRPO (cat. no 709-036-073, Jackson ImmunoResearch), Donkey anti-human IgM-HRPO (cat. no 709-036-098, Jackson ImmunoResearch).

Validation

All antibodies were validated by the relevant manufacturer.

Plants

Seed stocks

Not applicable.

Novel plant genotypes

Not applicable.

Authentication

Not applicable.

Flow Cytometry

Plots

Confirm that:

☒ The axis labels state the marker and fluorochrome used (e.g. CD4-FITC).

☒ The axis scales are clearly visible. Include numbers along axes only for bottom left plot of group (a 'group' is an analysis of identical markers).

☒ All plots are contour plots with outliers or pseudocolor plots.

☒ A numerical value for number of cells or percentage (with statistics) is provided.

Methodology

Sample preparation

Tissue specimens were mechanically dissociated using scissors followed by GentleMACs (Miltenyi Biotec) homogenization for 15 seconds in RPMI 1640, 10% FBS, 40 µg collagenase D (Roche) and 40 U/ml DNAse I (SIGMA-Aldrich). Samples were then incubated for 30 minutes at 37°C, followed by another 75 second homogenization cycle and passed through 70 µm cell strainer (Corning). Following a five min 930 x g centrifugation (Beckman Coulter, Allegra X-12R), the sample pellet was suspended in 5 ml and passed through a 40 µm cell strainer and centrifuged as before. Finally, the pellet was treated with 5 ml red blood cell lysis solution (QIAGEN) for 5 minutes, made up to 30 ml with PBS and centrifuged again. Cells were counted and split into equal numbers (1 - 5 million) to stain for flow cytometry. Lung draining lymph nodes were processed similarly without the need for GentleMACs homogenization or collagenase D and DNAse I treatment.
Isolated LMCs were processed for flow cytometry on the same day to avoid reduced viability following cryopreservation and thawing. To facilitate running matched blood samples on the same day as stained LMCs, isolated PBMCs were thawed on the day the lung samples were processed. Briefly, cryopreserved PBMCs were thawed, washed, and rested in RPMI 1640 containing 10% FBS for one hour in a 37°C, 5% CO2 incubator prior to staining. Between 1 - 5 million PBMCs or LMCs were stained with the respective B cell surface marker panels (Supplementary Tables 1 to 3) for 20 minutes at RT in the dark. Samples were washed twice with PBS and suspended in 250 µl 2% PFA-PBS and kept at 4°C in the dark. Samples were acquired on a BD FACS Aria Fusion III and data analyzed using FlowJo version 9.9.6 (Tree Star).

Instrument

Samples were acquired on a BD FACS Aria Fusion III.

Software

Data analyzed using FlowJo version 9.9.6 (Tree Star).

Cell population abundance

No sorting was done.

Gating strategy

Gating strategies are included in the manuscript for all relevant populations of interest.

☒ Tick this box to confirm that a figure exemplifying the gating strategy is provided in the Supplementary Information.
